# Donor/recipient ascending aortic diameter ratio as a novel potential metric for donor selection and improved clinical outcomes in heart transplantation: a propensity score-matched study

**DOI:** 10.3389/fcvm.2023.1277825

**Published:** 2023-10-25

**Authors:** Matiullah Masroor, Yuqi Chen, Yixuan Wang, Nianguo Dong

**Affiliations:** ^1^ Department of Cardiovascular Surgery, Union Hospital, Tongji Medical College, Huazhong University of Science and Technology, Wuhan, China; ^2^ Department of Cardiothoracic and Vascular Surgery, Amiri Medical Complex, Kabul, Afghanistan

**Keywords:** Donor/recipient ascending aortic diameter ratio, heart transplantation, donor selection, transplant recipients, size matching

## Abstract

**Background:**

Donor/recipient size matching is paramount in heart transplantation. Body weight, height, body mass index, body surface area, and predicted heart mass (PHM) ratios are generally used in size matching. Precise size matching is important to achieve better clinical outcomes. This study aims to determine the donor/recipient ascending aortic diameter (AAoD) ratio as a metric for donor selection and its effect on postoperative clinical outcomes in heart transplant patients.

**Methods:**

We retrospectively reviewed all consecutive patients who underwent heart transplantation from January 2015 to December 2018. A cutoff value of 0.8032 for the donor/recipient AAoD ratio (independent variable for the primary endpoint during unmatched cohort analysis) was determined for predicting in-hospital mortality. The patients were divided into two groups based on the cutoff value. Group A, AAoD < 0.8032 (*n* = 96), and Group B, AAoD > 0.8032 (*n* = 265). A propensity score-matched (PSM) study was performed to equalize the two groups comprising 77 patients each in terms of risk. A Cox regression model was developed to identify the independent preoperative variables affecting the primary end-point. The primary endpoint was all-cause in-hospital mortality.

**Results:**

A total of 361 patients underwent heart transplantation during the given period. On the multivariate analysis, donor/recipient PHM ratio [HR 16.907, 95% confidence interval (CI) 1.535–186.246, *P *= 0.021], donor/recipient AAoD ratio < 0.8032 (HR 5.398, 95% CI 1.181–24.681, *P *= 0.030), and diabetes (HR 3.138, 95% CI 1.017–9.689, *P* = 0.047) were found to be independent predictors of in-hospital mortality. Group A had higher 3-year mortality than Group B (*P *= 0.022). The surgery time was longer and postoperative RBC, plasma, and platelets transfusion were higher in Group A (*P *< 0.05). Although not statistically significant the use of continuous renal replacement therapy (*P* = 0.054), and extracorporeal membrane oxygenation (*P* = 0.086), was realatively higher, and ventilation time (*P* = 0.079) was relatively longer in Group A.

**Conclusions:**

The donor/recipient AAoD ratio is a potential metric for patient matching and postoperative outcomes in heart transplantation. A donor/recipient AAoD ratio > 0.8032 could improve post-heart transplantation outcomes and donor heart utilization.

## Introduction

Despite significant advances in the treatment of end-stage heart failure, heart transplantation (HTx) remains the definitive and effective therapeutic option. As of 2017, 144,230 cases of orthotopic heart transplantation have been registered worldwide (1). Due to the imbalance between donor supply and clinical demand, selecting a proper recipient and donor is of paramount importance in optimizing heart transplantation outcomes. Many available potential allografts may get discarded due to the strict donor selection criterion (2). Patients in need of HTx can benefit more when the characteristics of the donor and recipient are used simultaneously to determine the optimal donor/recipient match and make correct and fast organ allocation strategies. Matching donor and recipient organs according to donor-to-recipient weight, height, body mass index (BMI), body surface area (BSA), and predicted heart mass (PHM) ratios has already been performed (3). These size-matching metrics not only affect the outcomes of heart transplantation but also improve the efficacy of donor allocation.

Several donor and recipient variables have been linked to HTx prognosis. Donor age, left ventricular hypertrophy, history of diabetes mellitus, and graft's ischemic time increase the risk of recipient's death (2–6). In addition, older age, diabetes mellitus, high creatinine, reoperation, and prior ischemic cardiomyopathy in recipients reduce their survival rate (3, 7, 8).

The size of the aorta changes with age, body weight, and BSA (9). The aortic diameter may change as the circulatory demands of the body changes such as during pregnancy. Studies have shown that the aortic diameter of multiparous women was larger than that of uniparous and nulliparous women, indicating that the size of the aorta changes with increased circulatory demand and cardiac output (10). Therefore, the size of the aorta may reflect the total blood volume load and cardiac output, both of which can affect bodily perfusion. Therefore, people with different ascending aortic diameters (AAoDs) would have different volume loads and cardiac outputs. Transplanting a patient with a heart having a smaller AAoD than expected may deteriorate the outcomes, as a smaller AAoD may represent a heart that can handle a smaller volume load and has a lower cardiac output. We believe that the AAoD ratio may be a novel predictor that can affect postoperative outcomes in HTx, and therefore can be used as a metric for donor selection. No study discusses whether matching the AAoD of the donor to that of the recipient affects the clinical outcomes of HTx or if it is used as a metric for donor selection. In the current study, we tried to evaluate the effect of donor/recipient AAoD ratio on survival rate and other postoperative outcomes to optimize the donor selection strategy and improve clinical outcomes in HTx.

## Materials and methods

### Ethical statement

All donor grafts were donated to the Red Cross Society of Hubei Province and were allocated by the China Organ Transplant Response System. The study was approved by the ethics committee of Tongji Medical College of Huazhong University of Science and Technology (IORG No.: IORG0003571) and was performed in accordance with the national program for deceased-donor organ donation in China (11) (national protocol for China category I) (12). Written informed consent was obtained from all patients or their guardians. The clinical and research activities are consistent with the principles of the Declaration of Istanbul and the Declaration of Helsinki.

### Study population

**Figure 1:**
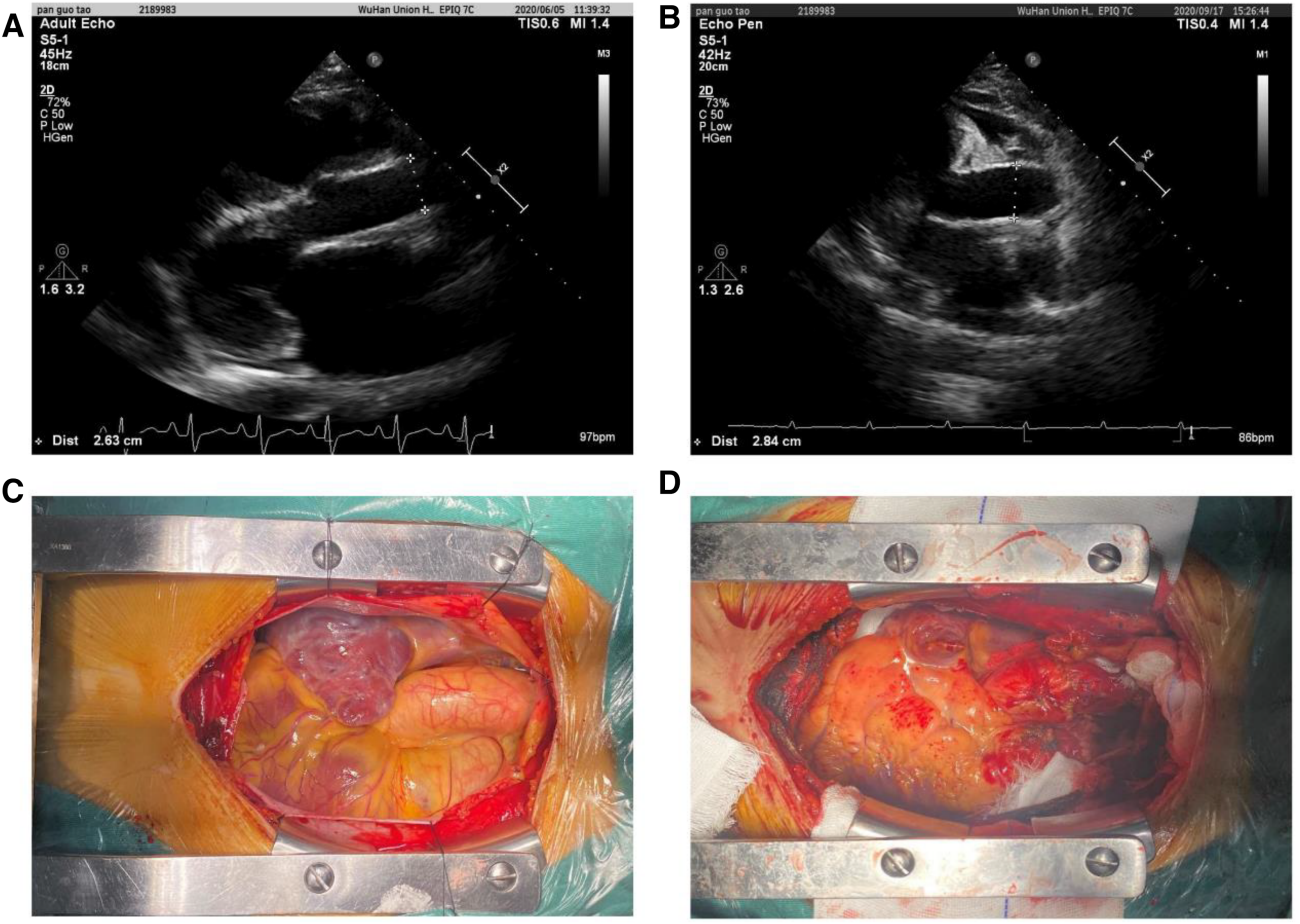
Ascending aortic diameters measured using the leading edge-to-leading edge convention in preoperative (**A**, recipient) and postoperative (**B**, donor) by two-dimensional echocardiography. Intraoperative AAo image before heart removal (**C**) and AAo image after donor heart implantation (**D**).

Our cohort incorporated all recipients of orthotopic heart transplantation at our institution from 1 January 2015 to 31 December 2018. A total of 361 patients were included, and all grafts were procured from donors after brain death. Patients who underwent cardiac retransplantation or multiorgan transplantation were excluded. Demographic and clinical characteristics of all heart transplant donors and recipients were collected retrospectively from electronic medical records, subsequent visits, or contact by our center. Preoperative echocardiography data were used to measure the recipient AAoD, and the donor AAoD was measured 3 weeks after operation (Figure 1). In this study, AAoD reflects the diameter of the ascending aorta 1 cm above the sinotubular junction (STJ). To obtain consistent data, we measured the AAoD at end-diastole using the leading edge-to-leading edge convention from the parasternal and suprasternal approaches by two-dimensional echocardiography.

### Organ preservation and operative technique

Donor heart removal was accomplished with *en bloc* removal of the superior vena cava with extra length of the inferior vena cava, aorta, and pulmonary artery. The organ was then prepared by marking the upper-left and lower-right pulmonary vein orifices and trimming the left atrium. A uniform preservation method was applied to all donor hearts during transport, consisting of 1 L of cold (4°C) histidine–tryptophan–ketoglutarate (HTK) solution. In addition, 500 ml of HTK solution was perfused before implantation.

### Immunosuppression

Interleukin-2 receptor antagonist (basiliximab) and methylprednisolone were used for induction therapy. The first dose of basiliximab (20 mg for children <35 kg, 40 mg for adults, and children ≥35 kg) was administered intravenously 2 h prior to the operation. The second dose was administered intravenously 4 days after transplantation. This medication was followed by a standard triple-drug immunosuppression regimen that included tacrolimus, mycophenolate mofetil, and prednisone. Maintenance immunotherapy consisting of tacrolimus (0–90 days, 10 ng/kg; 90 days–1 year, 8–10 ng/kg; >1 year, 5–8 ng/kg), mycophenolate (maintenance dose 1.5–2 g/day for adults, 600 mg/m^2^/day for children), and prednisone was used for all recipients. Following endomyocardial biopsy, acute cellular rejection exceeding grade 2R according to the ISHLT criteria (13) was treated by administering 500 mg of methylprednisolone for 3 days and increasing the dose of immunosuppressive drugs.

### Outcome measures

The primary endpoint was all-cause in-hospital mortality; mortality data were obtained from the China Heart Transplant Registration Network, where all deaths are registered, as required by law. 1 May 2019 was set as the endpoint of this study with a follow-up time of 37.1 ± 2.4 months for Group A and 44.9 ± 1.8 months for Group B. Secondary endpoints were the use of mechanical circulatory support (MCS), including an intra-aortic balloon pump, extracorporeal membrane oxygenation, and continuous renal replacement therapy (CRRT) in the early postoperative periods, and short-term postoperative complications, such as acute rejection, neurological complications, renal complications, hepatic dysfunction, and respiratory complications.

### Cutoff value of the donor/recipient AAoD ratio

**Figure 2:**
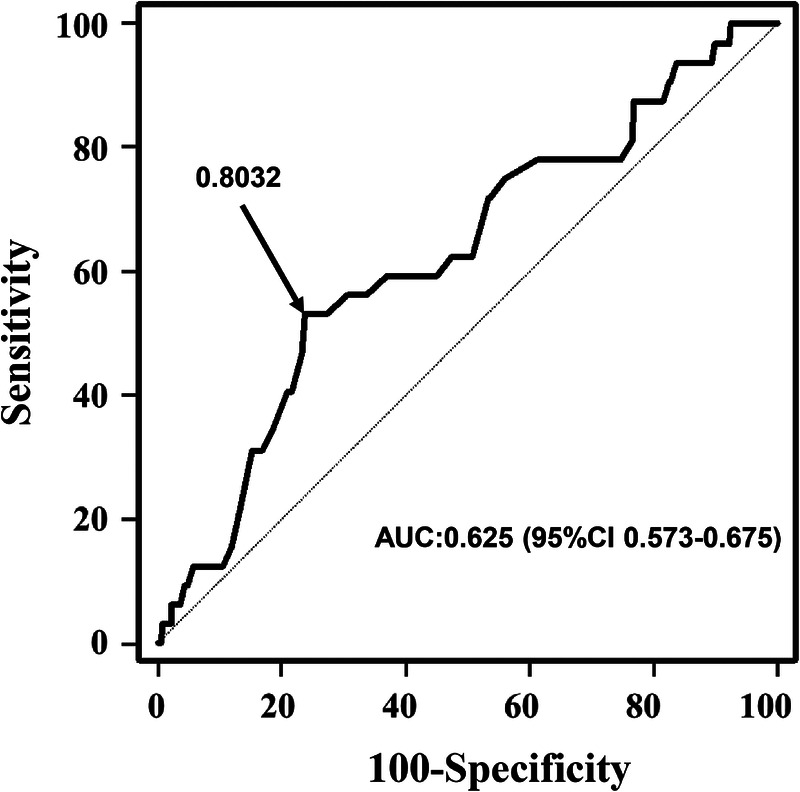
Cutoff value of donor/recipient ascending aortic diameter ratio for predicting in-hospital mortality by receiver operating characteristic curve in heart transplantation patients.

After univariate and multivariate analyses of the unmatched cohort that included all the variables listed in Table 1, we found that the donor/recipient AAoD ratio was an important independent statistically significant risk factor that affects postoperative survival. The receiver operating characteristic (ROC) curve was obtained to evaluate the cutoff value of AAoD for predicting all-cause mortality. The cutoff value was calculated corresponding to the maximum area under the ROC curve. The Youden index was used to identify the best cutoff value (*J* = sensitivity + specificity - 1). The ROC curve revealed a best cutoff value of 0.8032 [area under the curve (AUC) 0.625, 95% confidence interval (CI) 0.573–0.675] (Figure 2) for AAoD ratio.

**Table 1:** Preoperative characteristics of the overall cohort and subgroups divided based on the AAoD ratio.

Variable	Value (*n* = 361)	Group A < 0.8032 (*n* = 96)	Group B > 0.8032 (*n* = 265)	*P*-value
Age (years)	44.10 ± 16.31	53.19 ± 11.96	41.02 ± 16.63	**<0.001**
Weight (kg)	62.11 ± 16.27	65.11 ± 11.25	60.82 ± 17.50	0.101
BMI (kg/m^2^)	22.41 ± 8.79	23.31 ± 3.92	22.61 ± 10.34	0.576
Preoperative AAoD (cm)	3.04 ± 0.66	3.63 ± 0.50	2.83 ± 0.57	**<0.001**
Postoperative AAoD (cm)	2.79 ± 0.41	2.56 ± 0.31	2.87 ± 0.41	**<0.001**
Donor gender (male)	282 (87.11%)	75 (78.12%)	207 (78.11%)	0.679
Donor age (years)	32.64 ± 12.54	31.28 ± 12.94	33.46 ± 12.46	0.098
Donor weight (kg)	61.50 ± 12.21	59.95 ± 10.74	62.72 ± 12.72	0.090
Donor/recipient weight ratio	0.98 ± 0.36	0.94 ± 0.20	1.11 ± 0.39	**<0**.**001**
Donor/recipient PHM ratio	1.09 ± 0.29	1.02 ± 0.21	1.12 ± 0.32	**0**.**016**
Donor/recipient AAoD ratio	0.93 ± 0.22	0.71 ± 0.07	1.04 ± 0.18	**<0.001**
Cold ischemic time (min)	338.05 ± 112.17	342.81 ± 108.81	334.82 ± 115.47	0.496
Cold ischemic time (h)				0.973
0–4 (%)	59 (16.34%)	15 (15.62%)	44 (16.60%)	
4–6 (%)	128 (35.45%)	33 (34.37%)	95 (35.84%)	
6–8 (%)	150 (41.55%)	41 (42.70%)	109 (41.13%)	
>8 (%)	24 (6.64%)	7 (7.29%)	17 (6.41%)	
Recipient gender (male)	274 (75.90%)	71 (73.95%)	203 (76.60%)	0.560
Diagnosis				0.068
CM (%)	235 (65.09%)	52 (54.16%)	183 (69.05%)	
CAD (%)	61 (16.89%)	20 (20.83%)	41 (15.47%)	
VHD (%)	41 (11.35%)	18 (18.75%)	23 (8.67%)	
CHD (%)	15 (4.15%)	4 (4.16%)	11 (4.15%)	
Else (%)	9 (2.49%)	2 (2.08%)	7 (2.64%)	
Hypertension (%)	58 (16.06%)	29 (30.20)	29 (10.94)	**<0**.**001**
Diabetes (%)	47 (13.01%)	21 (21.87%)	25 (9.43%)	**0**.**002**
Hyperlipidemia (%)	14 (3.87%)	3 (3.12%)	10 (3.77%)	0.748
History of smoking (%)	129 (35.73%)	38 (39.58%)	89 (33.58%)	0.349
History of cardiac surgery (%)	94 (26.03%)	28 (29.16%)	66 (24.90%)	0.563
History of alcoholism (%)	84 (23.26%)	30 (31.25%)	54 (20.37%)	**0**.**027**
Peripheral vascular disease (%)	6 (1.66%)	2 (2.08%)	4 (1.50%)	0.723
Stroke (%)	10 (2.77%)	1 (1.04%)	9 (3.39%)	0.220
Dialysis (%)	4 (1.10%)	1 (1.04%)	3 (1.13%)	0.157
COPD (%)	10 (2.77%)	2 (2.08%)	7 (2.64%)	0.745
Chronic liver disease (%)	30 (8.31%)	8 (8.33%)	22 (8.30%)	0.969
Preoperative mechanical support (%)	8 (2.21%)	3(3.12%)	5(1.88%)	0.480

CM, cardiomyopathy; CAD, coronary artery disease; VHD, valvular heart disease; CHD, congenital heart disease.

Significant statistical values are in bold.

### Statistical analysis

Unless otherwise stated, the continuous variables conforming to a normal distribution were expressed as a mean ± standard deviation and analyzed with a two-sample *t*-test. The categorical variables were presented as numbers followed by percentages in parentheses and analyzed with the chi-squared test. To explore the influence of AAoD ratio on postoperative outcomes, the patients were divided into two groups based on the best cutoff value of the AAoD ratio: Group A (*n* = 96) with the donor/recipient AAoD ratio < 0.8032 and Group B (*n* = 265) with the donor/recipient AAoD ratio > 0.8032. Finally, propensity score matching was performed across key variables including data from the recipients and donors except AAoD and AAoD ratio to balance the distinction of baseline variables. A 1:1 match was performed with 0.02 tolerance and each group consisting of 77 patients. A univariate analysis for all-cause mortality was conducted using the Cox regression model. The candidate variables associated with a univariate effect on prognosis (*P*-value < 0.1) were then analyzed by stepwise multivariate regression with a probability of 0.05 and an elimination probability of 0.1. All tests were two-tailed with a significance level of *P* < 0.05. The time to event analysis was estimated by the Kaplan-Meier method and the difference between the groups by log-rank test. Statistical analysis was performed with SPSS Version 21.0 (IBM Corporation, Armonk, NY, USA) and MedCalc Version 19.0.4 (MedCalc Software, Ostend, Belgium).

## Results

### Baseline characteristics

From January 2015 to December 2018, 361 patients underwent heart transplantation in Wuhan Union Hospital. The baseline characteristics of the total cohort and PSM cohort are given in Table 1 and Table 2 respectively. After performing PSM, we found that the average age was 51.01 ± 12.42 years, with an average weight and BMI of 65.44 ± 12.62 kg and 24.16 ± 11.87 kg/m^2^, respectively. The number and percentage of patients with the following medical condition are as follows: hypertension, 39, 25.32%; diabetes, 26, 16.88%; hyperlipidemia, 9, 5.84%; history of smoking, 63, 40.91%; history of cardiac surgery, 45, 29.22%; history of alcohol use, 43, 27.92%; peripheral vascular disease, 5, 3.25%; stroke, 5, 3.25%; preoperative dialysis, 3, 1.95%; chronic obstructive pulmonary disease (COPD), 5, 3.25%; chronic liver disease, 9, 5.84%; and preoperative mechanical support, 5, 3.25%. The diagnoses for patients who required transplantation were cardiomyopathy (95, 61.69%), coronary artery disease (32, 20.78%), valvular heart disease (18, 11.69%), congenital heart defects (5, 3.25%), and others (4, 2.60%). A total of 134 donors were men (87.01%), and the average age and weight were 33.22 ± 12.63 years and 62.32 ± 11.36 kg, respectively. The standardized mean difference of all variables included in Table 1 and Table 2 is given in Supplementary Figure S1.

**Table 2:** Preoperative characteristics of the propensity score-matched cohort (*n* = 154).

Variable	Value (*n* = 154)	Group A < 0.8032 (*n* = 77)	Group B > 0.8032 (*n* = 77)	*P*-value
Age (years)	51.01 ± 12.42	52.15 ± 12.17	49.88 ± 12.64	0.470
Weight (kg)	65.44 ± 12.62	64.85 ± 11.18	66.04 ± 13.97	0.423
BMI (kg/m^2^)	24.16 ± 11.87	23.15 ± 3.93	25.17 ± 16.31	0.309
Preoperative AAoD (cm)	3.04 ± 0.66	3.18 ± 0.67	3.20 ± 0.47	0.781
Postoperative AAoD (cm)	2.79 ± 0.41	2.58 ± 0.33	2.97 ± 0.38	**<0.001**
Donor gender (male)	134 (87.01%)	66 (85.71%)	68 (88.32%)	0.632
Donor age (years)	33.22 ± 12.63	32.07 ± 13.12	34.36 ± 12.10	0.146
Donor weight (kg)	62.32 ± 11.36	60.99 ± 10.16	63.65 ± 12.38	0.544
Donor/recipient weight ratio	0.98 ± 0.24	0.96 ± 0.20	1.00 ± 0.27	0.751
Donor/recipient PHM ratio	1.04 ± 0.27	1.03 ± 0.21	1.04 ± 0.39	0.950
Donor/recipient AAoD ratio	0.93 ± 0.22	0.710 ± 0.070	0.987 ± 0.146	**<0.001**
Cold ischemic time (min)	343.13 ± 110.94	343.89 ± 110.92	342.36 ± 111.735	0.496
Cold ischemic time (h)				0.920
0–4 (%)	24 (15.58%)	12 (15.58%)	12 (15.58%)	
4–6 (%)	51 (33.12%)	24 (31.17%)	27 (35.06%)	
6–8 (%)	64 (41.56%)	34 (44.16%)	30 (38.96%)	
>8 (%)	15 (9.74%)	7 (9.09%)	8 (10.39%)	
Recipient gender (male)	125 (81.17%)	56 (72.73%)	69 (89.61%)	**0.007**
Diagnosis				0.173
CM (%)	95 (61.69%)	44 (57.14%)	51 (66.23%)	
CAD (%)	32 (20.78%)	14 (18.18%)	18 (23.38%)	
VHD (%)	18 (11.69%)	13 (16.88%)	5 (6.49%)	
CHD (%)	5 (3.25%)	4 (5.19%)	1 (1.30%)	
Else (%)	4 (2.60%)	2 (2.60%)	2 (2.60%)	
Hypertension (%)	39 (25.32%)	18 (23.38%)	21 (27.27%)	0.578
Diabetes (%)	26 (16.88%)	14 (18.18%)	12 (15.58%)	0.667
Hyperlipidemia (%)	9 (5.84%)	3 (3.90%)	6 (7.79%)	0.303
History of smoking (%)	63 (40.91%)	29 (27.66%)	34 (44.16%)	0.413
History of cardiac surgery (%)	45 (29.22%)	25 (32.47%)	20 (25.97%)	0.657
History of alcoholism (%)	43 (27.92%)	22 (28.57%)	21 (27.27%)	0.857
Peripheral vascular disease (%)	5 (3.25%)	2 (2.60%)	3 (3.90%)	0.649
Stroke (%)	5 (3.25%)	1 (1.30%)	4 (5.19)	0.173
Dialysis (%)	3 (1.95%)	3 (3.90%)	0 (0)	0.080
COPD (%)	5 (3.25%)	2 (2.60%)	3 (3.90%)	0.649
Chronic liver disease (%)	9 (5.84%)	6 (7.79%)	3 (3.90%)	0.303
Preoperative mechanical support (%)	5(3.25%)	3(3.90%)	2(2.60%)	0.649

Significant statistical values are in bold.

### Predictors of survival outcomes

We performed a univariate Cox regression analysis for the correlation between various preoperative variables and postoperative in-hospital mortality and found six variables that significantly affect the postoperative mortality (Table 3): BMI (HR 1.028, 95% CI 1.011–1.046, *P *= 0.001), donor weight (HR 1.045, 95% CI 1.007–1.085, *P *= 0.020), donor/recipient PHM ratio (HR 6.976, 95% CI 2.811–17.309, *P *< 0.001), donor/recipient AAoD ratio < 0.8032 (HR 3.918, 95%, CI 1.107–13.886, *P *= 0.018), diabetes mellitus (HR 3.961, 95% CI 1.472–10.658, *P *= 0.006), and COPD (HR 6.286, 95% CI 1.387–28.481, *P *= 0.017). After multivariate Cox regression analysis, only three independent variables were significant that affected the postoperative in-hospital mortality (Table 3), namely, donor/recipient PHM ratio (HR 16.907, 95% CI 1.535–186.246, *P *= 0.021), donor/recipient AAoD ratio < 0.8032 (HR 5.398, 95% CI 1.181–24.681, *P *= 0.030), and diabetes (HR 3.138, 95% CI 1.017–9.689, *P *< 0.047).

**Table 3:** Univariate and multivariate Cox regression analysis for predicting in-hospital mortality in the PSM heart transplantation cohort (*n* = 154).

Variable	Univariate analysis			Multivariate analysis		
	HR	95% CI	*P*-value	HR	95% CI	*P*-value
Age	1.037	0.992–1.084	0.105			
Weight	0.995	0.964–1.028	0.781			
BMI	1.028	1.011–1.046	**0** **.** **001**	0.994	0.956–1.032	0.741
Preoperative AAoD	1.369	0.640–2.928	0.419			
Postoperative AAoD	0.986	0.306–3.181	0.981			
Donor age	1.019	0.981–1.058	0.325			
Donor weight	1.045	1.007–1.085	**0**.**020**	1.029	0.977–1.085	0.281
Donor/recipient weight ratio	3.585	0.915–14.049	0.067			
Donor/recipient PHM ratio	6.976	2.811–17.309	**<0**.**001**	16.907	1.535–186.246	**0**.**021**
Donor/recipient AAoD ratio			** **			** **
<0.8032	3.918	1.107–13.886	**0**.**018**	5.398	1.181–24.681	**0**.**030**
>0.8032	Ref.					
Donor gender (male)	2.435	0.317–18.707	0.392			
Cold ischemic time (h)	0.061	0.290–1.027	0.546			
0–4 (%)	1.888	0.240–14.870	0.546			
4–6 (%)	0.505	0.051–4.982	0.559			
6–8 (%)	0.413	0.025–6.705	0.534			
>8 (%)	Ref.		0.134			
Recipient gender (male)	0.521	0.181–1.503	0.228			
Diagnosis	0.991	0.619–1.586	0.970			
CM (%)	0.227	0.028–1.822	0.163			
CAD (%)	0.055	0.003–0.899	0.062			
VHD (%)	0.107	0.007–1.749	0.117			
CHD (%)	0.168	0.009–3.002	0.226			
Else (%)	Ref.		0.987			
Hypertension	0.902	0.251–3.237	0.874			
Diabetes	3.961	1.472–10.658	**0**.**006**	3.138	1.017–9.689	**0**.**047**
History of Smoking	0.457	0.147–1.418	0.175			
History of cardiac surgery	0.759	0.292–1.972	0.571			
History of alcoholism	1.254	0.435–3.615	0.676			
Dialysis	0.044	0.000–5,792.297	0.604			
COPD	6.286	1.387–28.481	**0**.**017**	2.416	0.440–13.266	0.310
Chronic liver disease	2.489	0.553–11.194	0.235			
Preoperative mechanical support	3.088	0.401–23.758	0.279			

Significant statistical values are in bold.

### Short-term prognosis and outcomes of Group A and Group B

**Figure 3:**
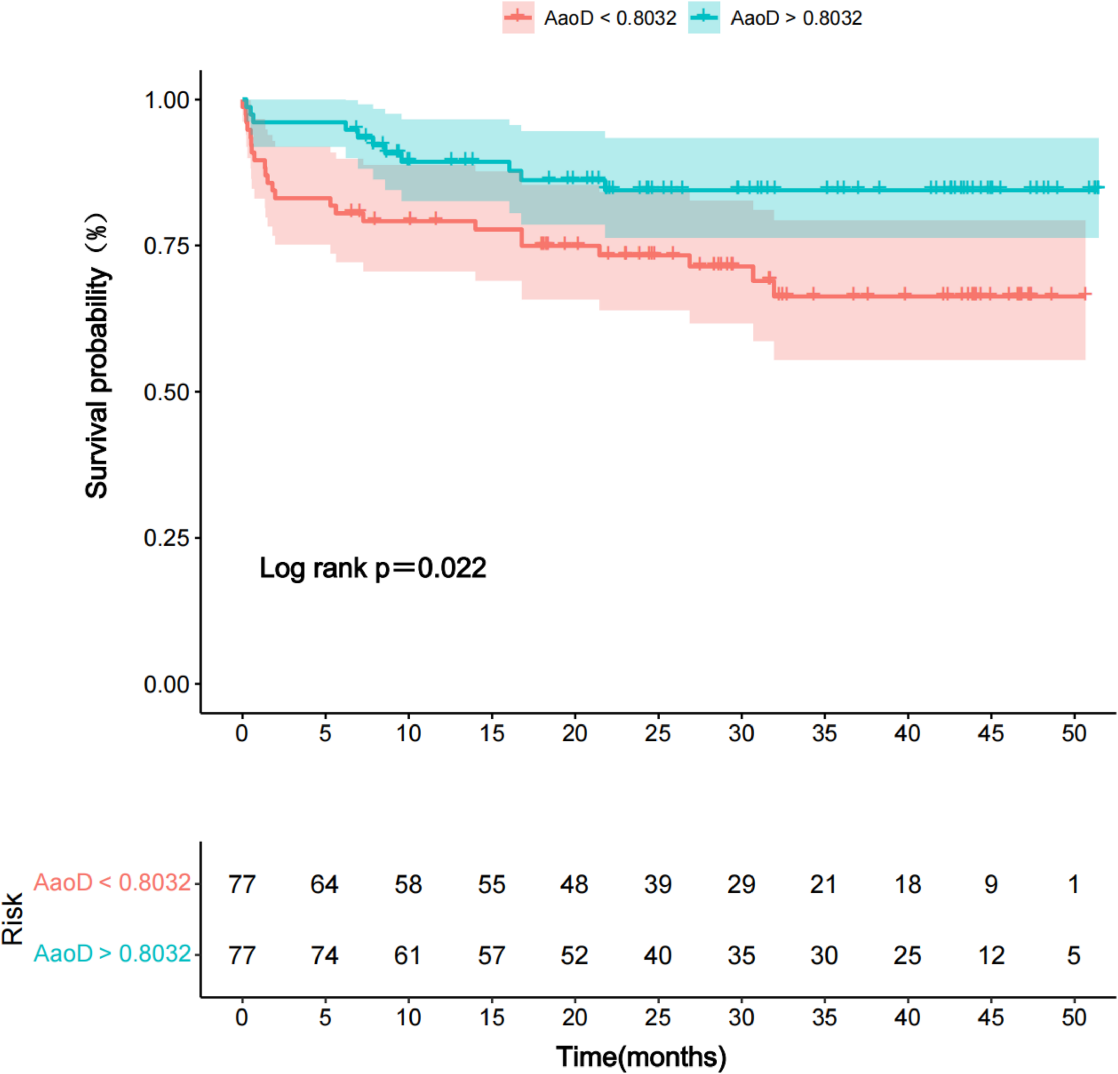
Kaplan–Meier survival curve comparing two groups: donor/recipient AAoD ratio <0.8032 (Group A) vs. >0.8032 (Group B).

The postoperative survival curve of the two groups is presented in Figure 3. The survival rate of Group B was significantly higher than that of Group A within the following time periods: 6 months (94.8%* *±* *2.5% vs. 80.5%* *±* *4.5%, *P *= 0.003) and 3 years (84.4%* *±* *4.4% vs. 66.3%* *±* *6.1%, *P *= 0.022) (Figure 3).

We then compared the postoperative outcomes of these two groups (Table 4). The results showed that the surgery time was longer in Group A than in Group B (257.33 ± 97.47 vs. 236.59 ± 56.67, *P* = 0.027). RBC transfusion (9.51 ± 13.33 vs. 7.48 ± 4.25, *P* = 0.021), plasma transfusion (797.08 ± 1,143.65 vs. 609.80 ± 273.32, *P* = 0.025), and platelets transfusion (2.96 ± 2.90 vs. 2.51 ± 1.52, *P* = 0.030) were significantly higher in Group A than in Group B. The number of patients who required postoperative dialysis in Group A was borderline statistically significantly higher than that in Group B (11, 14.47% vs. 4, 5.19%, *P *= 0.054). Meanwhile, even though not statistically significant the ECMO use (9.09% vs. 2.60%, *P* = 0.086) was relatively higher and ventilation time (5430.56 ± 11231.40 minutes vs. 3153.98 ± 2692.88 minutes, *P* = 0.079) was relatively longer in group A than in group B.

**Table 4:** Operative and postoperative outcomes of donor/recipient AAoD ratio <0.8032 (Group A) and >0.8032 (Group B).

Outcomes	Group A < 0.8032 (*n* = 77)	Group B > 0.8032 (*n* = 77)	*P*-value
CPB time (min)	110.38 ± 62.04	110.86 ± 42.73	0.425
Cross-clamping time (min)	32.56 ± 21.46	30.65 ± 8.87	0.951
Surgery time (min)	257.33 ± 97.47	236.59 ± 56.67	**0** **.** **027**
RBC transfusion (U)	9.51 ± 13.33	7.48 ± 4.25	**0**.**021**
Platelet transfusion (U)	2.96 ± 2.90	2.51 ± 1.52	**0**.**030**
Plasma transfusion (ml)	797.08 ± 1,143.65	609.80 ± 273.32	**0**.**025**
ICU length of stay (h)	302.30 ± 179.39	257.94 ± 75.53	0.121
IABP use (%)	28 (36.36)	25 (32.89)	0.652
ECMO use (%)	7 (9.09)	2 (2.60)	0.086
CRRT use (%)	11 (14.47)	4 (5.19)	0.054
24 h drainage volume (ml)	585.00 ± 987.04	437.65 ± 277.00	0.160
Re-thoracotomy (%)	4 (7.55)	1 (1.92)	0.176
Ventilation time (min)	5,430.56 ± 11,231.40	3,153.98 ± 2,692.88	0.079
Acute rejection (%)	1 (1.30)	0 (0)	0.319
Respiratory complications (%)	39 (60.94)	32 (50.79)	0.250
Inotropic support (%)	51 (96.23)	52 (100)	0.157
Dopamine (%)	51 (96.23)	52 (100)	0.157
Dobutamine (%)	38 (71.70)	32 (61.54)	0.270
Adrenalin hydrochloride (%)	45 (84.91)	47 (90.38)	0.394
Isoprenaline (%)	21 (39.62)	16 (30.77)	0.342
Neurological complications (%)	5 (7.81)	2 (3.17)	0.252
Renal complications (%)	2 (3.13)	2 (3.17)	0.987
Liver complications (%)	5 (7.81)	2 (3.17)	0.252
Positive sputum culture (%)	35 (45.45)	37 (48.05)	0.864
Positive blood culture (%)	6 (8.00)	7 (9.09)	0.810
LVEF (%)	59.58 ± 18.96	60.69 ± 18.45	0.055

IABP, intra-aortic balloon pump.

Significant statistical values are in bold.

## Discussion

The major novel findings of our study are as follows: the donor/recipient AAoD ratio is an important preoperative variable for in-hospital mortality in heart transplantation with a cutoff value of 0.8032. Survival analysis and secondary outcome comparison showed that donor/recipient AAoD ratio > 0.8032 results in a higher survival rate, low transfusion rate, shorter ventilation and surgery time, and less use of CRRT and ECMO. Thus, a donor/recipient AAoD ratio of >0.8032 may contribute to better survival and fewer postoperative complications in heart transplantation.

With the rapid development of HTx in China and worldwide, the imbalance between organ demand and donation is gradually emerging. More transplant recipients can benefit when donor characteristics are also used to determine the optimal donor/recipient match and make correct and fast organ allocation strategies. As listed in the 36th annual report of the ISHLT (1), the topic of donor/recipient size matching has gained much attention. Some researchers disagree with the traditional criteria for size matching as they believe heart transplantation can be performed safely when donors are undersized or the donor-to-recipient body weight ratio is low and can increase the donor pool (14–16). Other researchers have attempted to find the ideal metric for matching donor and recipient heart size by considering body weight, height, BMI, BSA, and PHM (3, 17–19).

As we know, weight, height, BSA, and BMI are all indirect ways to predict whether a heart size matches each other. So, there is always a search for new and more accurate indices to judge whether the donor heart anatomically and physiologically matches the recipient heart for heart transplantation. PHM is considered the most potent measure for size matching and predicting postoperative mortality. A study by Kransdorf et al. (3) assessed five heart size matching metric abilities, namely, body weight, height, BMI, BSA, and PHM, to predict 1-year mortality after heart transplantation. Recipients with undersized PHM were associated with higher 1-year mortality (HR 1.34, 95% CI 1.13–1.59, *P* < 0.001), while patients had no risk of increased mortality when donors were undersized for other metrics. They found that 32% of donors turned down for heart size would have been accepted if the PHM cutoff value of 0.86 was used. The 36th adult heart transplantation report of ISHLT (1) also suggests that using PHM as a donor/recipient size match may increase the available donor pool. The report also shows that PHM is modestly associated with body weight match and poorly associated with height match. In this study after multivariate analysis, the donor/recipient PHM ratio was an independent risk factor for in-hospital mortality (HR 16.907, 95% CI 1.535–186.246, *P *= 0.021), which is in accordance with previous studies. Another study by Gong et al. (20) compared the patients with implanted hearts undersized by >30% with those of implanted hearts matched for size (within 30%). They performed the analysis for undersize by total body weight (TBW) and undersize by PHM with a primary endpoint of moderate or severe primary graft dysfunction (PGD) within 24 h and a secondary endpoint of 1-year survival. PGD was associated with undersize by PHM (*P* = 0.007) but not TBW (*P* = 0.49), which was confirmed by multivariate analysis predicting that undersizing by PHM but not TBW is a predictor of PGD (OR* *=* *3.3, 95% CI* *=* *1.3–8.6). They concluded that size matching by PHM is clinically more appropriate than size matching by TBW (20).

The size of the recipient heart chambers and part of the ascending aorta changes immediately after transplantation, but other large blood vessels, body weight, and BSA remain the same. Therefore, the research is focused on these size-matching metrics that can predict whether the newly implanted heart best matches the explanted heart or adequately satisfies body perfusion demand. Some research studies have already been done on these size-matching metrics, as discussed above. In our study, we wanted to use the echocardiographic measurements of the AAoD of donors and recipients to predict the outcomes of HTx and attempted to find a new size-matching metric for donor selection. In our institution, donors were selected based on body weight (donor-to-recipient TBW* *>* *0.8) as reflected by the donor/recipient body weight ratio of 0.98 ± 0.36 in Table 1. After performing PSM, the multivariate analysis showed that except for donor/recipient PHM ratio (HR 16.907, 95% CI 1.535–186.246, *P *= 0.021), diabetes (HR 3.138, 95% CI 1.017–9.689, *P* = 0.047), and AAoD ratio (HR 5.398, 95% CI 1.181–24.681, *P *= 0.030) (which is the basis of our tow group categorization), other factors did not influence the survival. The similar risk groups, including similar donor/recipient weight ratio (*P* = 0.751), BMI (*P* = 0.309), recipient age (*P* = 0.470), and donor age (0.146) (Table 2), should have shown similar survival and clinical outcomes. Different survival and clinical outcomes of both groups indicate that the AAoD ratio may be a better indicator than other matching metrics such as body weight in HTx patients. Different outcomes of the two groups based on the AAoD ratio, even in weight-matched and other risk-matched patients, indicate that the AAoD ratio is a potential metric for clinical outcomes and should be considered during donor selection. In our univariate analysis, pretransplant (recipient) and posttransplant (donor) AAoD have also been listed in the risk factors, albeit not significant (*P *= 0.419 and *P *= 0.981, respectively). Simultaneous inclusion of donor and recipient data seems to be a better approach than taking the factors of the donor or recipient alone into account. We believe the AAoD ratio should be considered complementary rather than an alternative to already available size-matching metrics until further research clarifies these results.

Each donor heart during procurement retains a part of the ascending aorta for anastomosis during transplant. Therefore, by measuring the AAoD before and after the operation, we directly get the AAoD of the recipient and donor, respectively. AAoD might be influenced by the choice of measurement method, such as echocardiography or cardiac computed tomography. To obtain consistent data, we measured the AAoD 1 cm above STJ at end-diastole using the leading edge-to-leading edge convention from the parasternal and suprasternal approaches by two-dimensional echocardiography.

The size of the aorta changes with age, gender, body weight, and BSA (9, 21, 22). The natural compensatory mechanisms of the body would adjust the aortic size to best fit the perfusion demand of the body. The diameter of the aorta may decrease due to the atrophy of the aortic wall caused by low stroke volume and pulse pressure in heart failure patients who are candidates for HTx (23, 24). In addition, patients on MCS before HTx might have a smaller AAoD due to a loss of pulsatile pressure. Therefore, patients who are HTx candidates have a smaller AAoD compared with normal individuals of the same age, gender, and body weight, and AAoD at the time of HTx may not be the true representative of their volume load. AAoD trends with overall volume need and cardiac output, with a larger AAoD indicating a larger volume and cardiac output and vice versa (10). Similarly, a lower donor/recipient AAoD ratio also represents a relatively lower volume load and cardiac output of the body of the donor compared to that of the recipient. Therefore, taking a heart from a patient with a lower AAoD (representing lower volume and cardiac output) and placing it in a larger one will decrease the perfusion of the body. This decrease in body perfusion is due to a decrease in cardiac output. It is worth mentioning that the decrease in cardiac output may be due to a decrease in preload (a smaller AAoD probably represents a smaller heart) and an increase in afterload due to increased recipient volume load. We believe this may be the phenomenon behind worse outcomes in Group A patients compared to Group B patients. In the case of a lower donor/recipient AAoD ratio, after transplantation, the heart of the donor is exposed to a higher afterload, which could contribute to primary graft dysfunction (25).

A donor/recipient AAoD ratio of <0.8032 was associated with high mortality of HTx recipients not only in the hospital but also during the follow-up, along with notable postoperative complications during hospitalization. The surgery time was significantly longer in Group A. Group A had higher RBC, platelets, and plasma transfusion than Group B. Subramaniam et al. (26) reported that intra- and postoperative RBC transfusions (within the first 24 h) were associated with poor outcomes. The CRRT rate was relatively high in Group A; considering that both groups had similar rates of preoperative renal disease, the decrease in renal perfusion might have led to higher rates of CRRT use. The use of ECMO was also relatively high, which may indicate the effect of a lower donor/recipient AAoD ratio on heart function.

### Limitations

Our study has some limitations. The single-center, retrospective, and observational design limited the ability to correct all confounding factors and selection bias; however, we used PSM to match most of the confounders. Donor information was limited and needed to be further tracked and completed. Moreover, the specific mechanism of the AAoD ratio affecting prognosis remains uncertain, and the relationship between a lower AAoD ratio and preload and afterload/blood volume needs to be clarified in future studies. Finally, the follow-up time was not sufficient to evaluate long-term outcomes. High-quality, multicenter, large-sample prospective studies are required to further verify the conclusions.

In conclusion, HTx clinicians often face difficulty in donor selection, with the need to balance the risk of post-HTx mortality and worsening end-stage heart failure. The availability of a new potential variables facilitate the graft allocation and help in real-time decision-making. The donor/recipient AAoD ratio is a novel potential metric for donor selection and a ratio >0.8032 could improve donor heart utilization and post-HTx outcomes.

## Data Availability

The raw data supporting the conclusions of this article will be made available by the authors, without undue reservation.
