# Supplementary material for: Donor/recipient ascending aortic diameter ratio as a novel potential metric for donor selection and improved clinical outcomes in heart transplantation: a propensity score-matched study
Source: Front Cardiovasc Med. 2023 Oct 25;10:1277825. doi: 10.3389/fcvm.2023.1277825 (PMC10634287; doi:10.3389/fcvm.2023.1277825)
Supplement: Supplementary file 1 [file Image1.pdf]

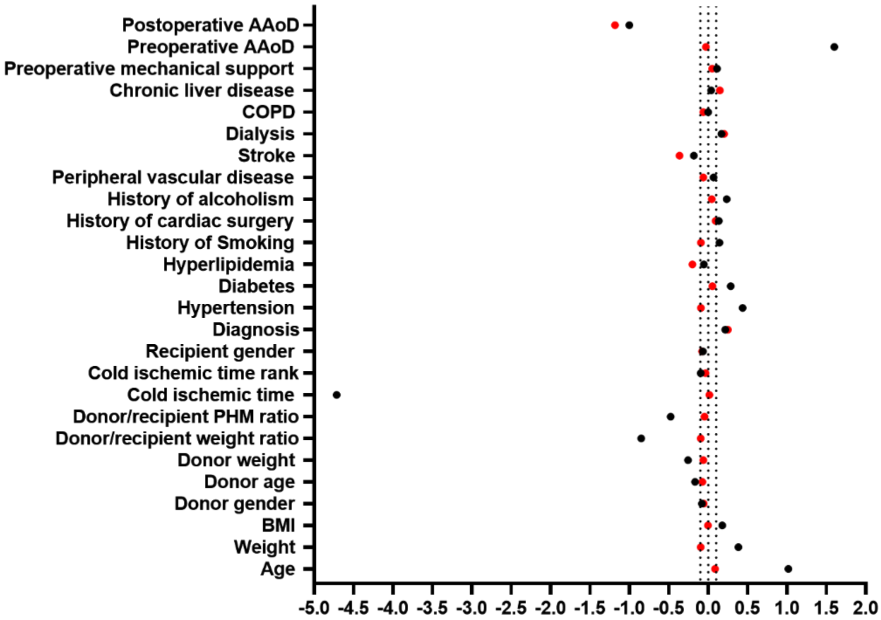

Supplementary Figure S1. Standardized mean difference of the variables included in Table 1 and Table 2.
